# Natural product-inspired synthesis of coumarin–chalcone hybrids as potential anti-breast cancer agents

**DOI:** 10.3389/fphar.2023.1231450

**Published:** 2023-09-06

**Authors:** Nabil A. Alhakamy, Mohammad Saquib, Sanobar, Mohammad Faheem Khan, Waseem Ahmad Ansari, Deema O. Arif, Mohammad Irfan, Mohammad Imran Khan, Mohd Kamil Hussain

**Affiliations:** ^1^ Department of Pharmaceutics, Faculty of Pharmacy, King Abdulaziz University, Jeddah, Saudi Arabia; ^2^ Mohamed Saeed Tamer Chair for Pharmaceutical Industries, Faculty of Pharmacy, King Abdulaziz University, Jeddah, Saudi Arabia; ^3^ Center of Excellence for Drug Research and Pharmaceutical Industries, King Abdulaziz University, Jeddah, Saudi Arabia; ^4^ Department of Chemistry, University of Allahabad, Prayagraj, India; ^5^ Department of Chemistry, Government Raza Post Graduate College, Rampur, India; ^6^ M. J. P. Rohilkhand University, Bareilly, India; ^7^ Department of Biotechnology, Era’s Lucknow Medical College, Era University, Lucknow, India; ^8^ Faculty of Medicine, Ibn Sina National College, Jeddah, Saudi Arabia; ^9^ Department of Medicine, Jawaharlal Nehru Medical College and Hospital, Aligarh Muslim University (AMU), Aligarh, India; ^10^ Departments of Biochemistry, Faculty of Science, King Abdulaziz University, Jeddah, Saudi Arabia; ^11^ Centre of Artificial Intelligence in Precision Medicine, King Abdulaziz University, Jeddah, Saudi Arabia

**Keywords:** breast cancer, coumarins, chalcones, neo-tanshinlactone, hybrid molecules

## Abstract

Twelve novel neo-tanshinlactone–chalcone hybrid molecules were constructed through a versatile methodology involving the Horner–Wadsworth–Emmons (HWE) olefination of 4-formyl-*2H*-benzo [*h*]chromen-2-ones and phosphonic acid diethyl esters, as the key step, and evaluated for anticancer activity against a series of four breast cancers and their related cell lines, *viz.* MCF-7 (ER + ve), MDA-MB-231 (ER-ve), HeLa (cervical cancer), and Ishikawa (endometrial cancer). The title compounds showed excellent to moderate *in vitro* anti-cancer activity in a range of 6.8–19.2 µM (IC_50_). Compounds **30 (**IC_50_ = 6.8 µM and MCF-7; IC_50_ = 8.5 µM and MDA-MB-231) and **31 (**IC_50_ = 14.4 µM and MCF-7; IC_50_ = 15.7 µM and MDA-MB-231) exhibited the best activity with compound **30** showing more potent activity than the standard drug tamoxifen. Compound **30** demonstrated a strong binding affinity with tumor necrosis factor α (TNF-α) in molecular docking studies. This is significant because TNFα is linked to MCF-7 cancer cell lines, and it enhances luminal breast cancer cell proliferation by upregulating aromatase. Additionally, virtual ADMET studies confirmed that hybrid compounds **30** and **31** met Lipinski’s rule; displayed high bioavailability, excellent oral absorption, favorable albumin interactions, and strong penetration capabilities; and improved blood–brain barrier crossing. Based on the aforementioned results, compound **30** has been identified as a potential anti-breast cancer lead molecule.

## 1 Introduction

**FIGURE 1:**
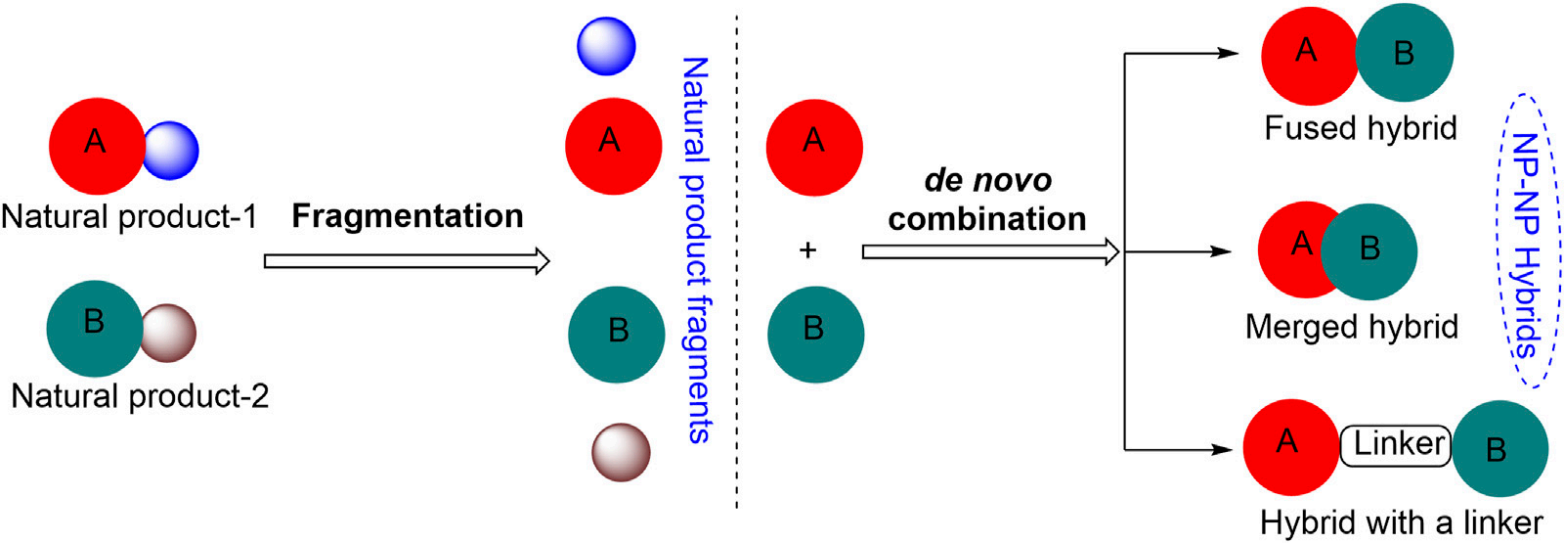
Approaches for the construction of small natural product hybrid molecules.

Breast carcinoma is the most commonly diagnosed cancer and the leading cause of death from cancer disease globally. In the year 2020, approximately 23 million new cases of breast cancer (11.7% of all cancer cases) were estimated worldwide (Siegel et al., 2020; Sung et al., 2021; Saquib et al., 2023). Despite the achievements and advances made in the chemotherapy of cancers during the past 20 years, the emergence of drug resistance continues to be a major issue in cancer treatment. Increasing prevalence of drug resistance combined with issues of tissue selectivity and toxicity in cancer chemotherapy has necessitated further research toward the development of less toxic and more effective therapeutic anti-breast cancer agents (Saquib et al., 2019). The search for small-molecule inhibitors (SMIs) based on novel molecular scaffolds that exhibit unique medicinal properties is largely inspired by biologically relevant molecules, primarily natural products (NPs). The structural diversity of NPs provides a rich source of molecular scaffolds that can be used as starting points for the design and discovery of new SMIs. By studying the structural features of NPs, researchers can identify new therapeutic agents with improved pharmacokinetic properties, increased potency, and reduced toxicity. However, the full potential of NPs in the design of new scaffolds for drug discovery is currently limited by the scarcity of guiding synthetic strategies available for this purpose. In this milieu, molecular hybridization can be a very useful tool (Li and Vederas, 2009; Wetzel et al., 2011). This approach involves combining two or more pharmacophoric units to obtain novel compounds with enhanced therapeutic properties and is finding increasing applications in the fields of drug discovery and medicinal chemistry (Meunier, 2008; Bosquesi et al., 2011; Saquib et al., 2013; Tukulula et al., 2013; Švenda et al., 2015) (Figure 1) (Tietze et al., 2003).

**FIGURE 2:**
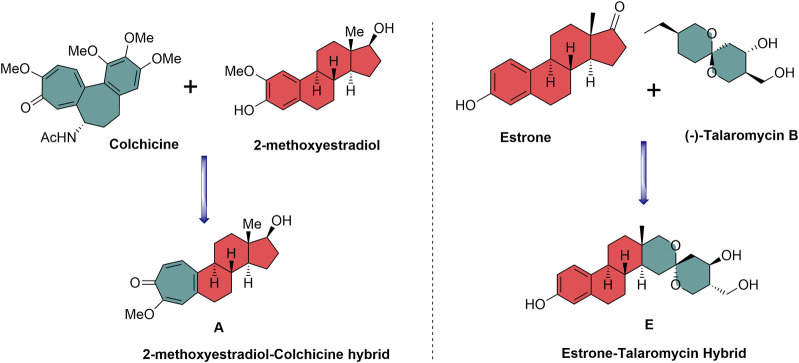
Design of biologically relevant natural product hybrids as anticancer agents.

2-Methoxyestradiol–colchicine hybrids and estrone–talaromycin hybrids are interesting examples of NP-based hybrid scaffolds. 2-Methoxyestradiol–colchicine hybrids were found to be more active as compared to the parent molecules against the tubulin polymerization assembly. 2-Methoxyestradiol–colchicine hybrid **A** inhibited polymerization of tubulin (IC_50_ = 2.1 µM) with greater potency as compared to parent molecules 2-methoxyestradiol (2-ME) (IC_50_ = 14.2 µM) and colchicine (IC_50_ = 11.2 µM) (Miller et al., 1997). The estrone–talaromycin hybrid (**B**), which was designed through the *de novo* combination of fragments of talaromycin B and estrone nucleus, exhibited better cytotoxic activity as compared to talaromycin B (Figure 2) (Tietze et al., 1998).

Several naturally occurring and synthetic coumarin derivatives have been reported to exhibit potent anti-cancer activity including anti-breast cancer activity (Musa et al., 2008; Küpeli Akkol et al., 2020). Neo-tanshinlactone, a benzocoumarin isolated from the underground stem of *Salvia miltiorrhiza*, inhibited the proliferation of MCF-7 cell lines more effectively than the anti-breast cancer drug tamoxifen (Wang et al., 2004; Lin et al., 2016). Likewise, chalcone is an important pharmacophore widely distributed in many natural products. Various natural and synthetic chalcones have been reported to show excellent biological activities including anticancer activity (Malek et al., 2011; Zhang et al., 2013; Zhuang et al., 2017). Moreover, a naturally occurring coumarin–chalcone hybrid isolated from the leaves of *Cyclosorus parasiticus* have been found to be cytotoxic (Quadri-Spinelli et al., 2000; Wei et al., 2013).

**FIGURE 3:**
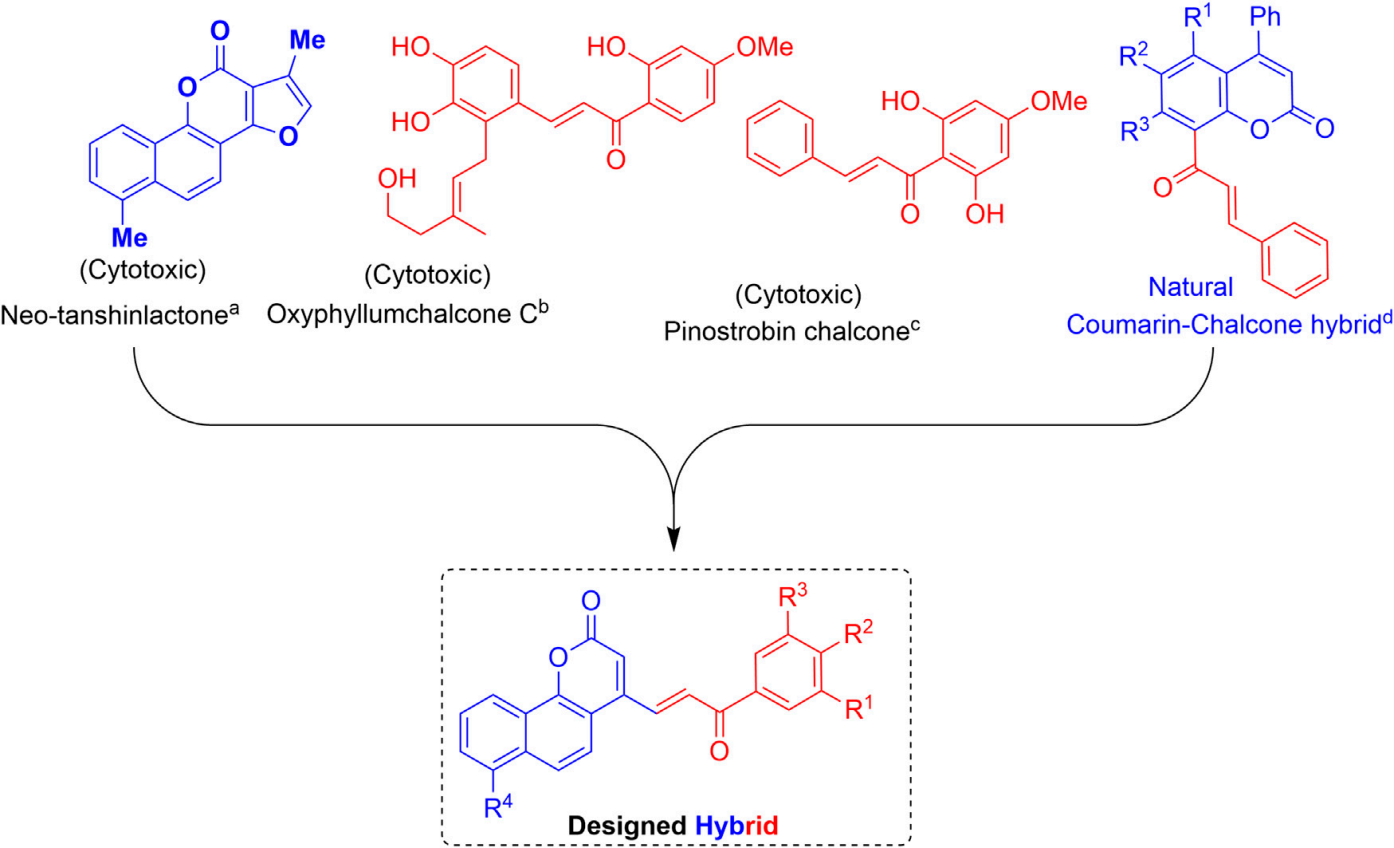
Basis of work: combination of neo-tanshinlactone and the chalcone units to obtain neo-tanshinlactone–chalcone hybrids. (a) (Wang et al., 2004); (b) (Zhang et al., 2013); (c) (Malek et al., 2011); and (d) (Quadri-Spinelli et al., 2000; Wei et al., 2013).

The interesting pharmacological properties of the neo-tanshinlactone and the chalcone scaffolds inspired us to design the neo-tanshinlactone–chalcone hybrid molecules (coumarin–chalcone hybrids) by the *de novo* combination of both these scaffolds onto one template (Figure 3).

## 2 Results and discussion

**FIGURE 4:**
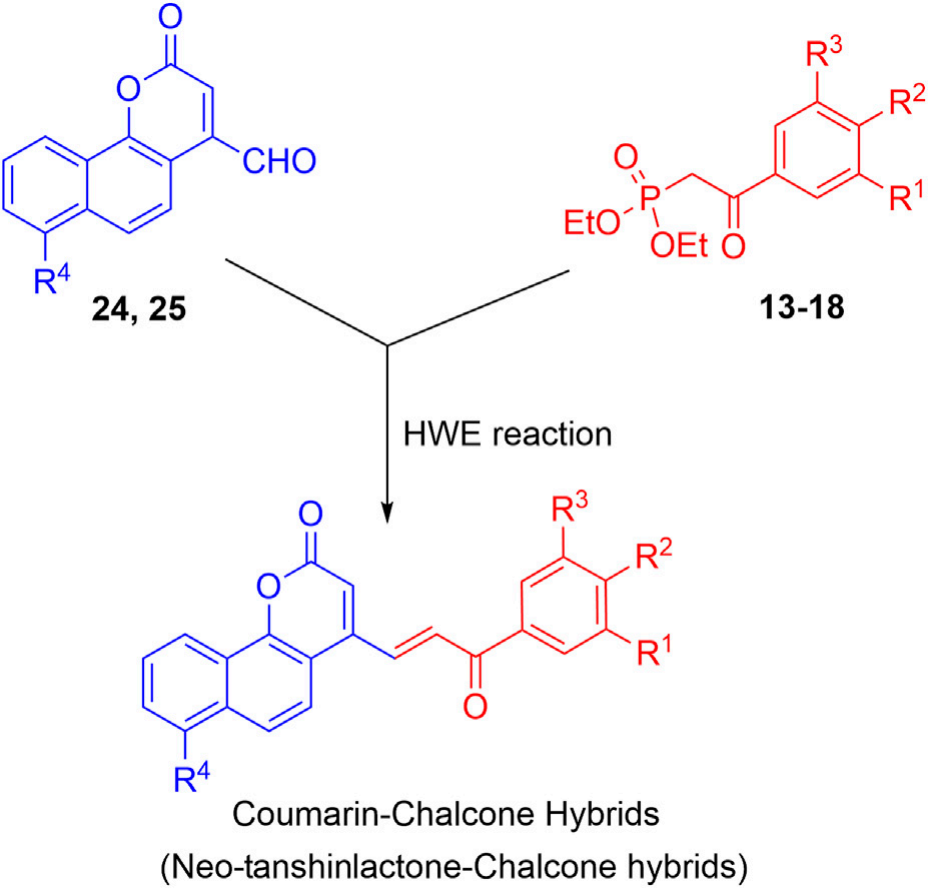
Outline for the synthesis of neo-tanshinlactone–chalcone hybrids.

**FIGURE 5:**
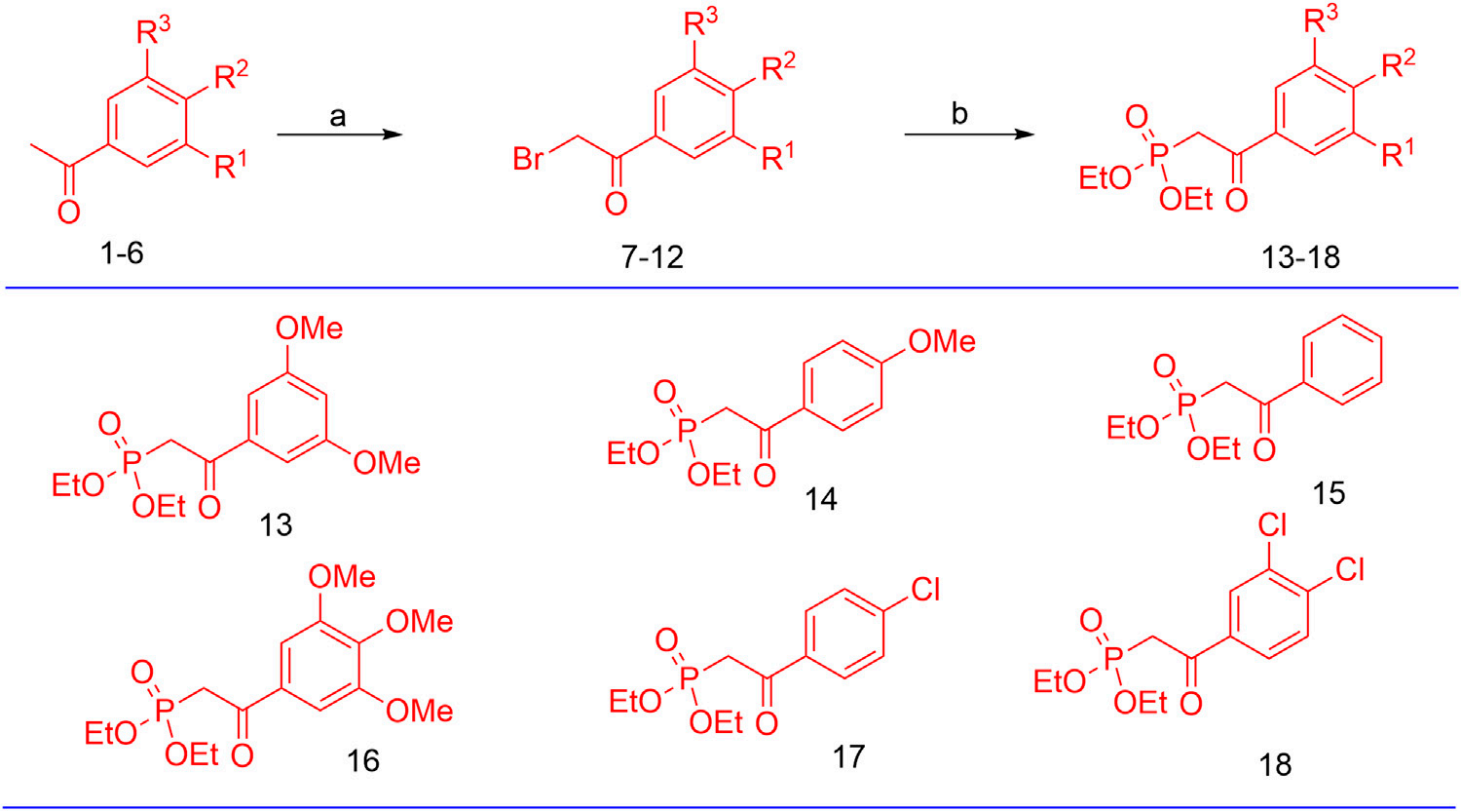
Preparation of *β*-aryl-*β*-ketophosphonates (**13–17**) through the Michaelis–Arbuzov reaction; reagent and conditions: (a) Br_2_, Et_2_O, and (b) P(OEt)_3_.

**FIGURE 6:**
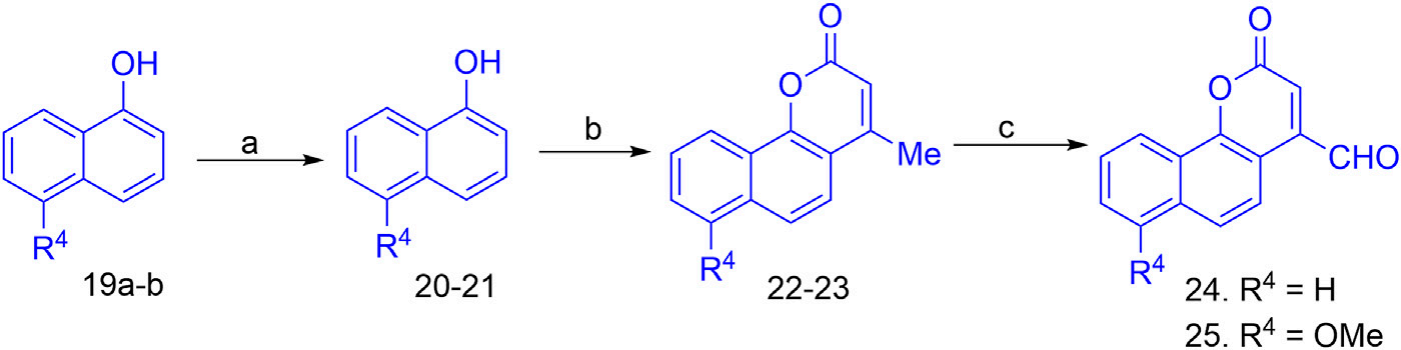
Reagent and conditions for the synthesis of 4-formyl-*2H*-benzo [*h*]chromen-2-ones; (a) MeI (1equiv), anhyd. K_2_CO_3_, acetone; (b) Cat conc. H_2_SO_4_, RT; (c) SeO_2_, xylene, reflux.

The synthesis of the target hybrid molecules was envisaged by the coupling of *β*-aryl-*β*-ketophosphonates (**13–17**) with the 4-formyl-2*H*-benzo [*h*]chromen-2-ones (**24**–**25**) (Figure 4) using the Horner–Wadsworth–Emmons (HWE) reaction, a versatile and efficient synthetic tool for the synthesis of diverse types of chalcone molecules and stilbene derivatives. The HWE reaction would allow us to synthesize a library of the target chalcones in quantitative yields. *β*-aryl-*β*-ketophosphonates (**13**–**17**) were prepared by the Michaelis–Arbuzov reaction of substituted phenacyl bromides and triethylphoshphite (Figure 5) (Debrouwer et al., 2013; Dunny et al., 2013; Saquib et al., 2021). 4-Formyl-2*H*-benzo [*h*]chromen-2-ones, **24**–**25**, on the other hand, were synthesized by the Riley oxidation of 4-methyl-2*H*-benzo [h]chromen-2-ones, **22**–**23**, in quantitative yields, which, in turn, were synthesized in excellent yields through an acid-catalyzed Pechmann condensation of ethyl acetoacetate (EAA) and 2-naphthols, **20–21**, at room temperature (Figure 6).

**FIGURE 7:**
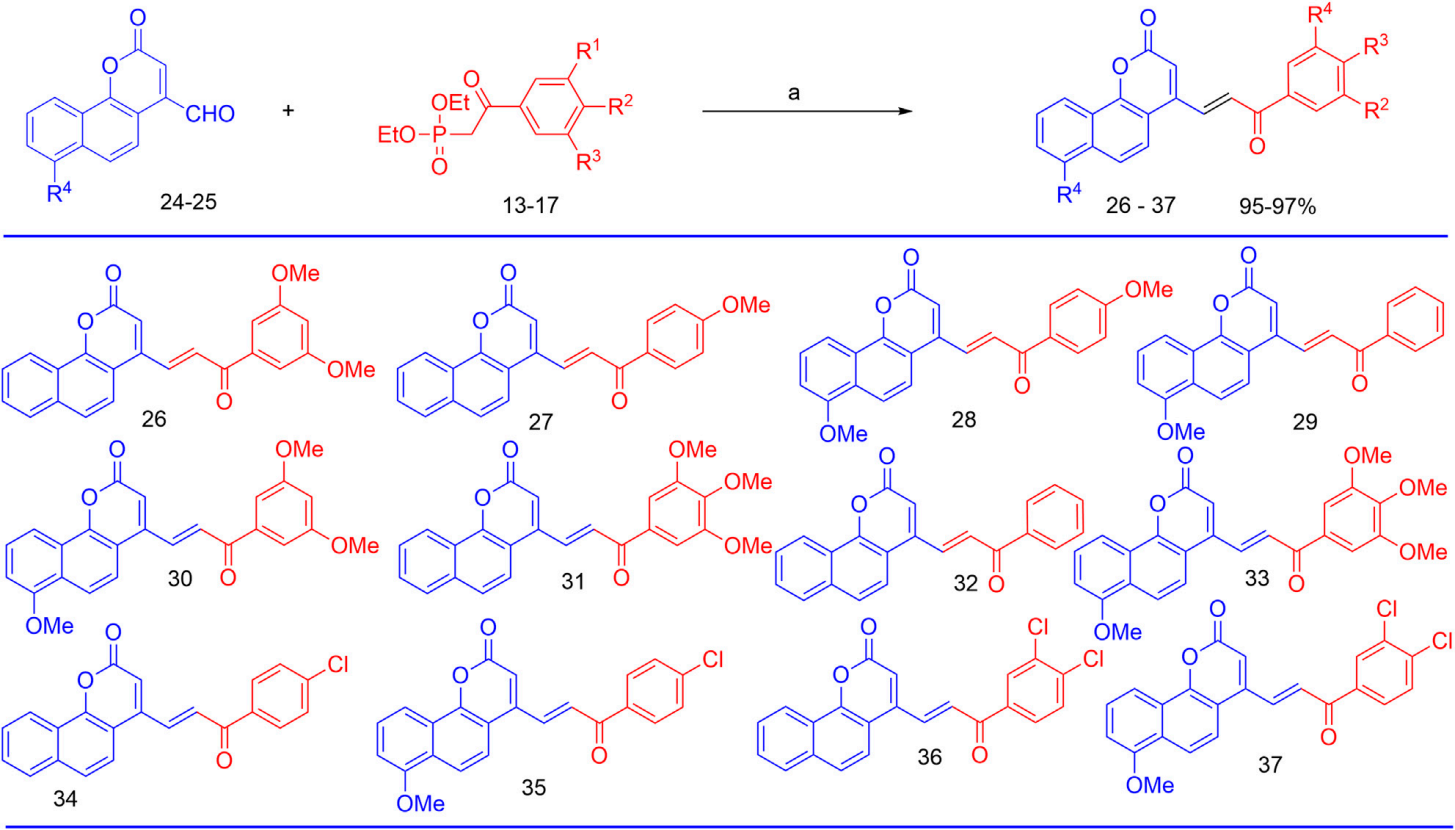
Reagents and conditions for the synthesis of neo-tanshinlactone–chalcone hybrids; (a) NaOMe, Anhyd DMF, and RT.

Finally, the olefination of β-aryl-β-ketophosphonates, **13**–**17**, with 4-formyl-2H-benzo [h]chromen-2-ones, **24**–**25**, in dry DMF/MeONa led to the synthesis of 2H-benzo [h]chromen-2-ones–chalcone hybrids (neo-tanshinlactone–chalcone hybrids), **26–37**, in excellent yields (95%–97%) (Figure 7). The synthesized compounds **26–37** were purified by recrystallization using ethyl acetate. We confirmed the structures of these compounds by NMR spectroscopy, mass spectrometry, and elemental analysis.

## 3 *In vitro* antiproliferative activities of the synthesized compounds

We conducted an *in vitro* MTT assay to evaluate the anti-cancer activity of the synthesized neo-tanshinlactone–chalcone hybrid compounds, **26**–**37**, against four human cancer cell lines, namely, MCF-7 (breast cancer), MDA-MB-231 (breast cancer), Ishikawa (endometrial cancer), and HeLa (cervical cancer). The toxicity of the hybrid molecules on the normal human embryonic kidney cell line HEK-293 was also evaluated.

**FIGURE 8:**
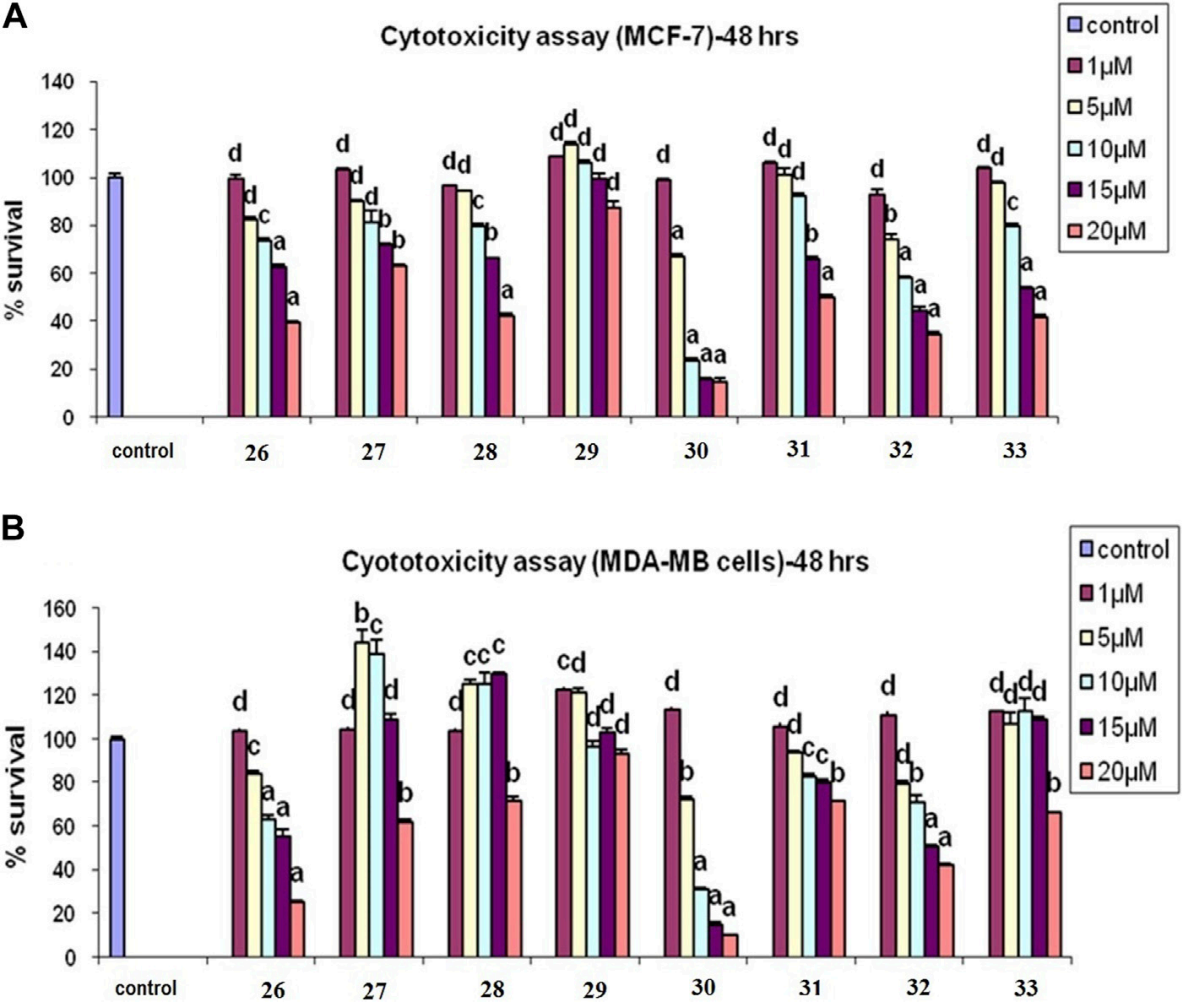
**(A)** Cytotoxicity assay of compounds **26**–**33** in MCF-7 cells. **(B)** Cytotoxicity assay of **26**–**33** in MCF-7 cells in MDA-MB-231 cells: results are expressed as mean ± SE; n = 5; *p* values are a-p<0.001, b-p<0.01, c-p<0.05, and d > 0.05.

From among the evaluated molecules, four compounds, i.e., **26**, **30**, **31**, and **33**, showed anti-cancer activity against MCF-7 cell lines. These compounds had methoxy substituents at the 3′, 5′, and 3′, 4′, and 5′ positions of the aroyl rings. Additionally, compounds **26**, **30**, and **31** also exhibited anti-cancer activity against MDA-MB-231 cell lines. Among these, **30** showed the highest anti-cancer activity against both MCF-7 and MDA-MB-231 cell lines with IC_50_ values of 6.8 µM and 8.5 µM, respectively (Table 1; Figures 8A,B). However, these compounds did not show activity against Ishikawa and HeLa cell lines (IC_50_ > 20 µM).

**TABLE 1:** *In vitro* anti-cancer activity and receptor-binding affinity of hybrid compounds, 26–37, against various cancer cell lines.

S. No.	Comp. no.	IC_50_ values (mean ± SEM, in μM)				
		MCF-7	MDA-MB-231	Ishikawa	HeLa	HEK-293
1	**26**	**17.2 ± 1.75**	**16.6 ± 1.25**	>20	>20	>20
2	27	>20	>20	17.4 **±** 2.72	>20	>20
3	28	**18.6 ± 2.30**	>20	>20	>20	>20
4	29	>20	>20	>20	>20	>20
5	30	**6.8 ± 1.48**	**8.5 ± 1.12**	ND	ND	>20
6	31	**14.4 ± 1.68**	**15.7 ± 1.01**	>20	>20	>20
7	32	>20	>20	ND	ND	>20
8	33	**16.3 ± 2.30**	**>**20	>20	>20	>20
9	34	>20	>20	>20	>20	>20
10	35	>20	>20	>20	>20	>20
11	36	>20	>20	19.2 ± 3.12	16.8 ± 3.14	>20
12	37	>20	>20	18.6 ± 2.56	17.2 ± 2.88	>20
13	TAM	11.2 ± 2.36	15.5 ± 2.64	ND	ND	18.8 ± 2.22

The bold values represent the IC_50_ values of compounds showing significant anti-cancer activity.

Similarly, compound **31** also exhibited potent inhibitions against MCF-7 and MDA-MB-231 cell lines with IC_50_ values of 14.4 µM and 15.7 µM, respectively, while compound **26** exhibited anti-cancer activity with IC_50_ values of 17.2 µM and 16.6 µM, respectively, in both of these cell lines. Molecules **34**, **35**, **36**, and **37** having chloro substituents on the aroyl ring were found inactive against MCF-7 and MDA-MB-231 cell lines (IC_50_ > 20 µM); however, compounds **36** and **37** showed good antiproliferative activity against Ishikawa and HeLa cell lines. Molecule **36** showed anti-cancer activity against Ishikawa and HeLa cell lines and with IC_50_ values of 19.2 µM and 16.8 µM, respectively, while compound **37** exhibited inhibition against Ishikawa and HeLa cell lines with IC_50_ values of 18.6 µM and 17.2 µM, respectively. Molecules **29** and **32** having unsubstituted aroyl rings were found inactive (IC_50_ > 20 µM) against all the four cancer cell lines. The results of the assay showed that the synthesized compounds had significant anti-cancer activity against all the four cell lines.

Interestingly, all the evaluated compounds were found to be non-toxic to normal HEK-293 cells with an IC_50_ value greater than 20 µM. This result indicates that the synthesized compounds, particularly compounds **30** and **31**, could be good candidates for further lead optimization.

## 4 Analysis of molecular docking

**FIGURE 9:**
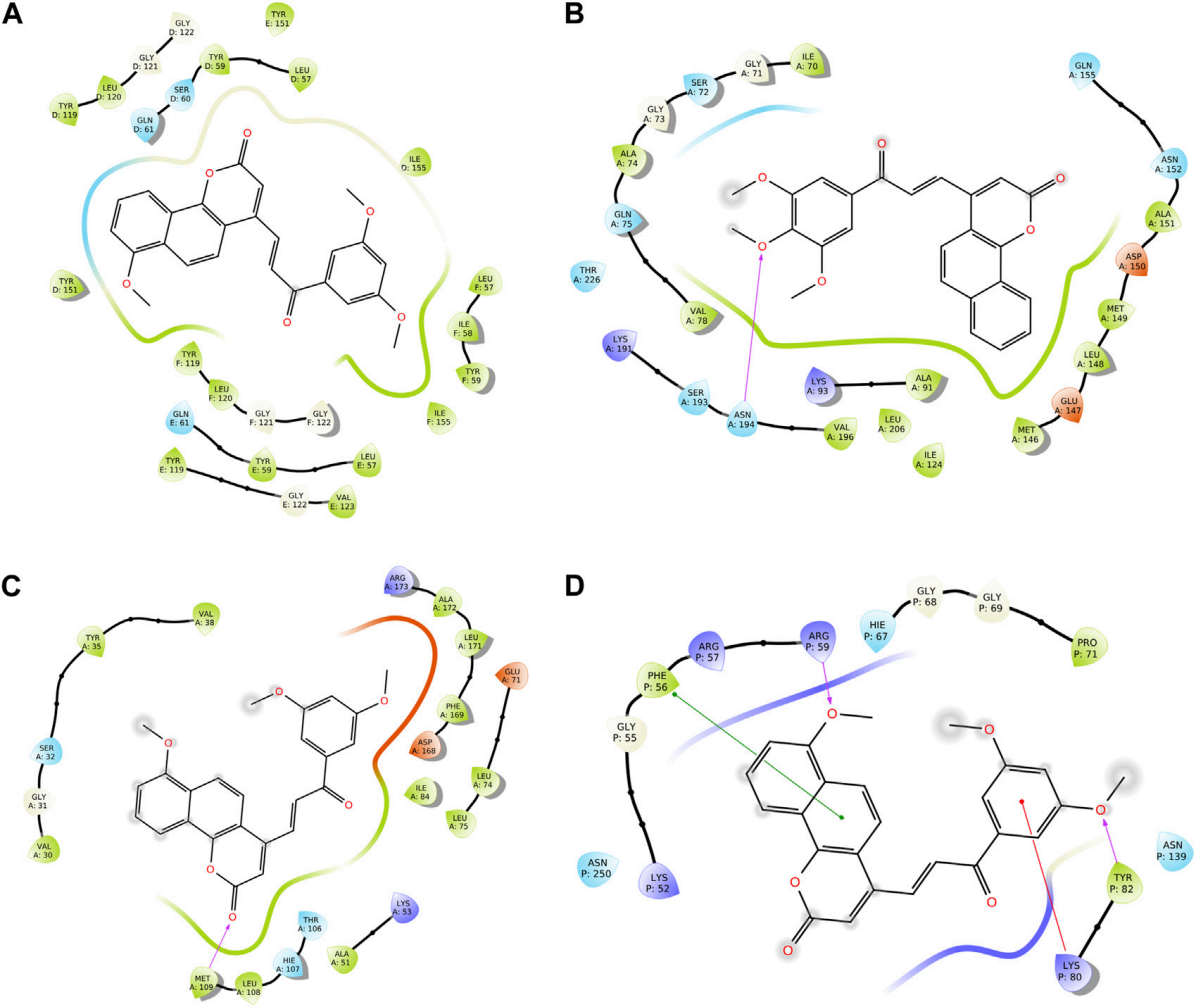
Two-dimensional (2D) views of molecular docking: **(A)** molecular interactions of compound **30** within the pockets of the TNF-α target, **(B)** molecular interactions of compound **31** within the pockets of the JNK target, **(C)** molecular interactions of **30** within the pockets of the MAPK target, and **(D)** molecular interactions of compound **30** within the pockets of the NF-kB target.

**FIGURE 10:**
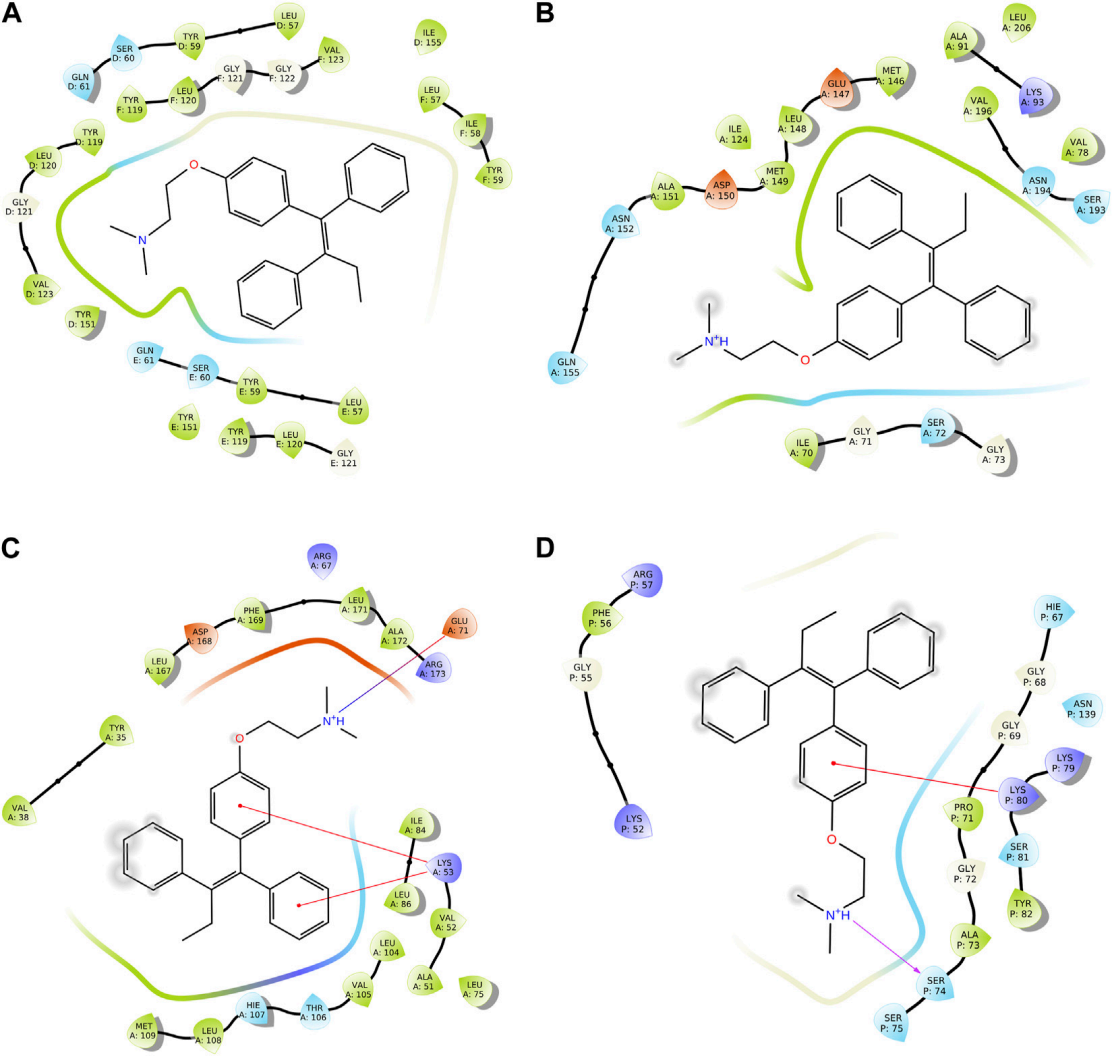
Two-dimensional (2D) views of molecular docking: **(A)** molecular interactions of tamoxifen within the pockets of the TNF-α target, **(B)** molecular interactions of tamoxifen within the pockets of the JNK target, **(C)** molecular interactions of tamoxifen within the pockets of the MAPK target, and **(D)** molecular interactions of tamoxifen within the pockets of the NF-kB target.

We conducted a molecular docking study for all the synthesized compounds, **26–37**, to investigate their binding interactions with the target proteins of cancer cell lines. The target proteins such as tumor necrosis factor α (TNF-α), c-Jun N-terminal kinase (JNK), mitogen-activated protein kinase (MAPK), and nuclear factor-kB (Nf-kB) were selected based on their involvement in cell proliferation, which is a key hallmark of cancer. The docking scores of the compounds provided an estimate of their binding affinity to the target proteins. This study can help us understand how these compounds interact with cancer cells and thus potentially lead to the development of new cancer agents. The docking scores of the compounds are shown in Table 2. The binding affinities of all docked compounds were in complete agreement with the obtained *in vitro* results and showed that compounds **30** and **31** exhibited potent inhibitory activities against TNF-α, JNK, MAPK, and Nf-kB targets. Considering the effect of hydrogen bonds, hydrophobic, electrostatic, and pi–pi interactions, the docking results revealed that all the compounds, **6**–**37**, displayed significant docking scores ranging from −7.508 to −10.170 kcal/mol against the TNF-α target, −6.867 to −7.516 kcal/mol against the JNK target, −3.936 to −5.285 kcal/mol against the MAPK target, and −3.257 to −4.507 kcal/mol against the NF-kB target, respectively (Mendie and Hemalatha, 2022). Compound **30** demonstrated polar and hydrophobic interactions with Ser60 and Gln61, and Leu57, Ile58, Tyr119, Leu120, Tyr151, and Ile155 amino acids of TNF-α protein (Figure 9A). Similarly, compound **30** displayed H-bonding with Met109, and polar and hydrophobic interactions with Ser32, Thr106, and Hie107, and Val30, Tyr35, Val38, Ala51, Leu74, Leu75, Ile84, Leu108, Phe169, Leu171, and Ala172 amino acids of the MAPK target (Figure 9C). Compound **30** also exhibited H-bonding with Arg59 and Tyr82 along with pi–pi, pi–cation, polar, and hydrophobic interactions with Phe56, Lys80, Hie67, Asn139, Asn250, and Pro71 amino acids of the NF-kB target (Figure 9D). On the other hand, compound **31** showed H-bonding with Asn109, and polar and hydrophobic interactions with Ser72, Gln75, Asn152, Gln155, Ser193, Thr226, Ile70, Ala74, Val74, Ala91, Met146, Leu148, Met149, and Ala151 amino acids of JNK protein (Figure 9B). Additionally, tamoxifen (control drug) exhibited polar and hydrophobic interactions with Ser60 and Ser61, and Leu57, Ile58, Tyr59, Tyr119, Leu120, Val123, Tyr151, and Ile155 amino acids of TNF-α protein (Figure 10A). Moreover, tamoxifen also showed polar and hydrophobic interactions with Ser72, Asn152, Gln155, Ser193, and Asn194, and Ile70, Val78, Ala91, Ile124, Leu148, Met149, and Ala151 amino acids of the JNK target (Figure 10B). Tamoxifen showed salt bridge, pi–cation, and hydrophobic interactions with Glu71 and Lys53, and Tyr35, Val38, Ala51, val52, Leu75, Ile84, Leu86, Leu104, Val105, Leu108, Met109, Leu167, Phe169, Leu171, and Ala172 amino acids of MAPK protein (Figure 10C). Tamoxifen also displayed H-bonding, pi–cation, polar and hydrophobic interactions with Ser74, Lys80, Hie67, Ser75, and Asn139, and Phe56, Pro71, Ala73, and Tyr82 amino acids of NF-kB protein (Figure 10D) (Guerrero-Perilla et al., 2015).

**TABLE 2:** Docking scores of compounds, 26–37, against the target proteins which are involved in cell proliferation.

Comp no.	TNF-α (6X81)	JNK (1JNK)	MAPK(1A9U)	NF-KB (1SVC)
26	−9.148	−7.048	−4.90	−3.504
27	−9.116	−6.945	−4.769	−4.199
28	−7.452	−6.932	NB	−3.976
29	−7.529	−7.055	−4.006	−3.736
**30**	**−10.170**	−6.966	**−5.285**	**−4.507**
31	−7.771	**−7.516**	−3.936	−3.608
32	−9.527	−7.074	−5.219	−4.429
33	−7.086	−6.913	−5.284	−4.879
34	−9.649	−7.491	−5.007	−4.324
35	−7.779	−6.867	−5.154	−3.257
36	−9.719	−7.014	−4.815	−4.003
37	−7.508	−6.704	−4.878	−3.192
Tamoxifen	−6.809	−6.544	−4.987	−3.162

The bold values represent the IC_50_ values of compounds showing significant anti-cancer activity.

## 5 Analysis of molecular dynamic simulation

**FIGURE 11:**
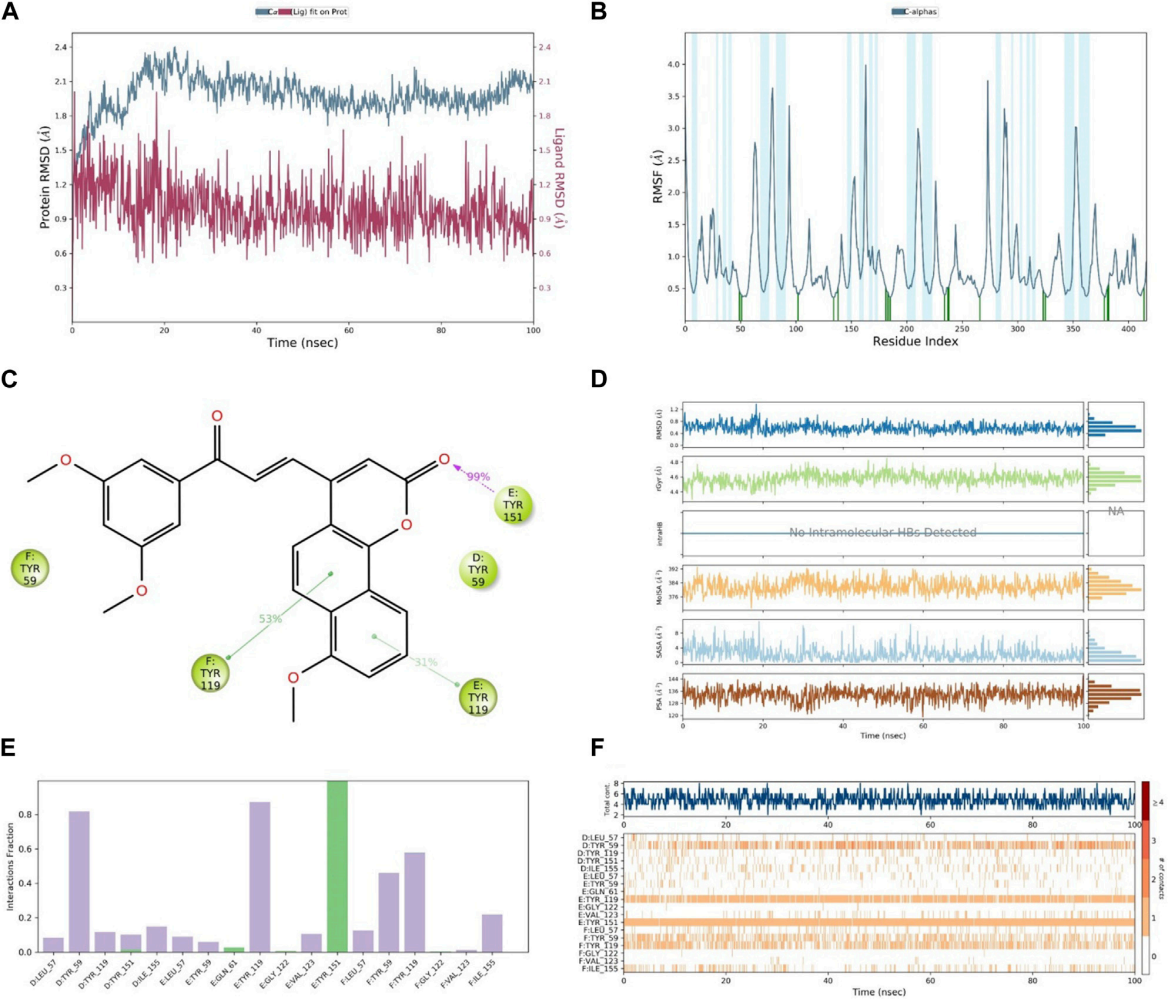
**(A)** RMSD of compound 30 (red) and TNF-α (gray), **(B)** RMSF of TNF-α, **(C)** contact of compound 30 with residues, **(D)** properties of the ligand, **(E)** interactions of compound 30 with residues via the bar chart, and **(F)** histogram of the no. of contacts between the ligand and amino acids of TNF-α.

After the analysis of the *in vitro* and docking studies, we found that compound **30**, as the most active derivative of neo-tanshinlactone, inhibited the cell proliferation of cancer cells via the suppression of the TNF-α, MAPK, and Nf-kB signaling pathways. Because of the highest binding, we performed MD simulation on compound **30** to study the stability within binding pockets of TNF-α target protein. A 100-ns trajectory was used to extract out RMSD, RMSF, RyG, and the number of hydrogen bonds of the ligand–protein complex by applying the OPLS3 force field in the molecular system. In this experiment, we found that the RMSD value of the ligand varies from 0.512 Å to 2.010 Å (Table 3A) and it exhibited the conformational rotation in the active site of the protein. The RMSD value of the protein varies from 1.162 Å to 2.402 Å (Table 3). Initially, it showed an RMSD value 2.402 for 20 ns time and then attained the equilibrium throughout the simulation. The RMSF value of Cα protein deviated from 0.363 Å to 3.987 Å (Table 3A), but the residue Asn39 from the chain E fluctuated to 3.987 Å, which is attributed to the stability for the whole simulation (Khan et al., 2022). In order to optimize the ligand properties, **30** was found to deviate up to 18.30 ns, which had shown RMSD values in the range of 0.62 Å to 1.38 Å and then attained equilibrium at 0.41 Å. The rGyr value of the ligand was calculated from 4.295 Å to 4.850 Å, followed by the achievement of the equilibrium point at 4.5 Å. Similarly, MolSA, SASA, and PSA were computed for the ranges 369.444 Å^2^–393.097 Å^2^, 0.000 Å^2^–11.171 Å^2^, and 118.929 Å^2^–146.046 Å^2^. As shown in Figure 11, these properties of compound **30** showed that it remains stable throughout the MD simulation. The protein–ligand interactions of the stable **30**-TNF-α complex were analyzed by a histogram, as shown in Figure 11F. The protein–ligand interactions were distinguished by including hydrogen, ionic, and hydrophobic bonds, and water bridges. We observed that compound **30** showed five hydrogen bonds with chain D: Tyr151, chain E: Gln61, chain E: Gly122, chain E: Tyr151, and chain F: Gly122 amino acids of the TNF-α target (Table 4). On the other hand, throughout the MD simulation, it formed a very stable intermolecular hydrogen bond with chain E: Tyr151, which contributed to more than 99% stability of the compound **30**-TNF-α complex. Despite the hydrogen bonds, compound **30** also showed 14 hydrophobic interactions with chain D: Leu57, chain D: Tyr59, chain D: Tyr119, chain D: Tyr151, chain D: Ile155, chain E: Leu57, chain E: Tyr59, chain E: Tyr119, chain E: Val123, chain F: Leu57, chain F: Tyr59, chain F: Tyr119, chain F: Val123, and chain F: Ile155 residues (Table 3B) (Abdalla et al., 2021; Kumar et al., 2022).

**TABLE 3:** A) MD simulation parameters of the 30-TNF-α complex and B) ligand interaction with amino acids of TNF-α (PDB: 6X81.

**A**	**Parameter**	**Target receptor**
	RMSD of the ligand (Å)	0.512–2.010
	RMSD of Cα (Å)	1.162–2.402
	RMSF of Cα protein (Å)	0.363–3.987
	rGyr (Å^2^)	4.295–4.850
	MolSA (Å^2^)	369.444–393.097
	SASA (Å^2^)	0.000–11.171
	PSA (Å^2^)	118.929–146.046

**B**		
	H-bonds	D: Tyr151, E: Gln61, E: Gly122, E: Tyr151, and F: Gly122
	Hydrophobic bonds	D: Leu57, D: Tyr59, D: Tyr119, D: Tyr151, D: Ile155, E: Leu57, E: Tyr59, E: Tyr119, E: Val123, F: Leu57, F: Tyr59, F: Tyr119, F: Val123, and F: Ile155

**TABLE 4:** Pharmacological parameters of compounds 26–37.

Parameter	26	27	28	29	30	31	32	33	34	35	36	37
**MW**	386.4	356.3	386.4	356.3	416.4	416.4	326.3	446.4	360.7	390.8	395.20	425.26
**HBD**	-	-	-	-	-	-	-	-	-	-	-	-
**HBA**	6	5	6	5	6	6	4	7	4	5	4	5
**PSA**	82.53	74.41	81.24	73.06	89.38	89.49	66.20	96.44	66.20	73.06	66.22	73.07
**LogP**	3.85	3.78	3.90	3.84	3.97	4.01	3.72	4.12	4.23	4.34	4.67	4.78
**LogS**	−5.07	−4.88	−5.16	−5.00	−5.32	−5.28	−4.74	−5.57	−5.51	−5.77	−6.15	−6.41
**Caco cell**	785.81	784.88	785.84	786.10	786.69	786.00	785.49	786.42	785.47	786.08	784.31	784.94
**logBB**	−1.01	−0.91	−1.01	−0.92	−1.11	−1.09	−0.82	−1.20	−0.67	−0.77	−0.55	−0.65
**MDCK cell**	381.24	380.75	381.25	381.39	381.70	381.34	381.07	381.56	939.63	940.41	1956.63	1958.24
**HSA**	0.249	0.27	0.26	0.29	0.24	0.26	0.29	0.25	0.42	0.41	0.54	0.53
**HOA**	3	3	3	3	3	3	3	3	3	3	3	3
**%HOA**	100	100	100	100	100	100	100	100	100	100	100	100

^a^ **MW**, molecular weight (130–725); **HBD**, hydrogen bond donor (0–6); **HBA**, hydrogen bond acceptor (2–20); **PSA**, polar surface area (7–200Å2); **LogP**, lipophilicity (−2.0–6.5); **Log S**, solubility (−6.5-0.5 mol/dm3); **Caco cell**, colon cancer cell (<25 poor and >500 high); **LogBB**, brain barrier (−3.0–1.2); **MDCK cell**, Madin–Darby canin kidney cell (<25 poor an >500 high); **HSA**, human serum albumin (−1.5–1.5); **HOA**, human oral absorption (1 for low, 2 for medium, and 3 for high); **%HOA**, percentage of human oral absorption.

## 6 Pharmacological parameters of compounds

Preliminary screening of the hybrid compounds showed that none of the compounds violated the rule of five (Lipinski’s rule), and all of them showed high bioavailability. Consequently, we studied the pharmacological properties of compounds **30** and **31**, which showed the highest molecular docking score, in detail. Both the compounds exhibited 100% human oral absorption and good interactions with serum albumin 0.24 and 0.26 within the range (−1.5 to +1.5), respectively. Furthermore, they demonstrated high penetration capabilities with values of 786.69 nm/s and 786.00 nm/s against Caco, and 381.70 nm/s and 381.34 nm/s against MDCK cells, respectively. Moreover, compounds **30** and **31** showed a significantly improved capability to cross the blood–brain barrier with values of −1.11 and −1.09, which were lower than the specified criterion from −3.0 to 1.2 (Table 4). Considering the aforementioned results, we can say that compounds **30** and **31** have all the attributes to be potent therapeutic agents (Kumar et al., 2023)**.**

## 7 Critical analysis, current study limitations, and future perspective

Although the aforementioned strategy enabled us to synthesize a diverse library of the target hybrid molecules, we could not synthesize coumarin–chalcone hybrids containing phenolic rings in the chalcone motif using the HWE strategy. The synthesis of the phenolic ring containing hybrid molecules may have been possible if the Claisen–Schmidt reaction was used instead of the present strategy. However, our efforts to couple the β-aryl-β-ketophosphonates with the 4-formyl-2H-benzo [h]chromen-2-ones to obtain the target hybrid molecules using the Claisen–Schmidt reaction were not successful. Considering the potential usefulness of the coumarin–chalcone hybrids as novel anti-breast cancer compounds, we are now concentrating our efforts toward finding a new synthetic protocol which will enable us to synthesize newer derivatives of the aforementioned hybrid molecules and evaluate their anti-breast cancer activity in our search for more potent and less toxic anti-breast cancer agents.

## 8 Conclusion

In summary, a library of 12 coumarin–chalcone hybrid compounds, **26**–**37**, were constructed, based on the molecular hybridization concept, via a practical and effective synthetic route which involved the HWE coupling of 4-formyl-2H-benzo [h]chromen-2-ones with phosphonic acid diethyl esters. The *in vitro* anti-cancer activity of the synthesized hybrid molecules was evaluated using an MTT assay, wherein they were found to exhibit potent to moderate anti-cancer activity against a panel of four cancer cell lines, including two breast cancer (MCF-7, ER + ve and MDA-MB-231, ER-ve), one cervical cancer (HeLa), and one endometrial cancer (Ishikawa) cell lines. Among these, molecules **30** and **31** were found to be the most potent, with compound **30** demonstrating superior activity against both ER + ve (IC_50_ = 6.8 µM, MCF-7) and ER-ve (IC_50_ = 8.5 µM, MDA-MB-231) breast cancer cell lines as compared to the standard drug tamoxifen. The toxicity of the molecules, **26**–**37**, was also evaluated on the normal human embryonic kidney cell line HEK-293, and interestingly, none of the screened molecules exhibited any toxicity against normal HEK-293 cells. Compound **30** also demonstrated a strong binding affinity with the tumor necrosis factor α (TNF-α) in molecular docking studies. This is significant because TNFα is associated with MCF-7 cancer cell lines. Virtual ADMET studies validated the compliance of hybrid compounds **30** and **31** with Lipinski’s rule. Moreover, they exhibited high bioavailability, excellent oral absorption, favorable albumin interactions, strong penetration capabilities, and improved blood–brain barrier crossing, indicating their potential as potent therapeutic agents. Thus, compound **30** could be considered a potential anti-breast cancer lead molecule, possibly by targeting TNFα.

## 9 Experiments

### 9.1 Chemistry

All the chemicals were procured from Across Organics and Sigma-Aldrich, and were used without further purification. The IR spectra of the compounds were recorded using a Perkin–Elmer Spectrum GX FTIR spectrometer. ^1^H NMR and ^13^C NMR spectra were recorded using the Bruker DRX-300 (300 MHz for ^1^H and at 75 MHz for ^13^C) or DPX-200 (at 50 MHz for ^13^C) spectrometer using CDCl_3_, DMSO-*d*_6_, or TFA-d_1_ (see the Supporting Information). Chemical shifts (δ) are reported in parts per million (ppm), using TMS as an internal standard. ESI–MS spectra were recorded using an LCQ advantage ion-trap mass spectrometer (Finnigan Thermo Fisher Scientific), and high-resolution mass spectra (ESI–HRMS) were recorded using an Agilent 6520 ESI-QTOP mass spectrometer.

#### 9.1.1 General procedure for the synthesis of 2-bromo-1-(aryl) ethan-1-ones (7–12)

A solution of bromine (7.50 mmol; 1.0 equiv), dissolved in Et_2_O (15 mL), was added to the stirred solution of the respective substituted acetophenones (7.5 mmol) in Et_2_O (15 mL) in a 50-mL round bottom flask at 0°C, and the mixture was stirred for 2 h at room temperature. After the completion of the reaction, it was quenched with the saturated NaHCO_3_ solution (100 mL) and extracted with Et_2_O (3 × 40 ml). The combined organic layers were dried over anhydrous Na_2_SO_4_ and concentrated *in vacuo* to obtain the crude mixture which was subsequently crystallized from EtOH to obtain the pure compounds **9** and **10**. Compounds **7**, **8**, **11**, and **12**, on the other hand, are commercially available.

**2-Bromo-1-(3,5-dimethoxyphenyl)ethan-1-one (9)**: white crystal (87%); ^1^H NMR (400 MHz; CDCl_3_), 7.24 (d, *J* = 2.6 Hz, 2H), 6.78 (d, *J* = 2.4 Hz, 1H), 4.79 (s, 2H), and 3.89 (s, 6H).
**2-Bromo-1-(3,4,5-trimethoxyphenyl)ethan-1-one (10)**: white crystals (85%); ^1^H NMR (400 MHz; CDCl_3_), 7.18 (s, 2H), 4.37 (s, 2H), 3.88 (s, 3H), and 3.84 (s, 6H).

#### 9.1.2 General procedure for the synthesis of diethyl 2-oxo-2-arylethylphosphonates (13–18)

Respective 2-bromo-1-(aryl)-ethan-1-ones (**7–12**, 5 mmol) and triethylphoshphite (40 mmol) were dissolved in acetonitrile (10 mL) using a round bottom flask and heated to 80°C for 2 h. After completion of the reaction, DCM (15 mL) and water (30 mL) were added to the reaction mixture, and it was extracted with DCM (3 × 40 mL). The combined organic layers were dried using Na_2_SO_4_ and concentrated *in vacuo*. The crude residue so obtained was purified through column chromatography to yield pure phosphonates (**13–18**). The analytical data on the phosphonates were found to be in agreement with those reported in the literature (Debrouwer et al., 2013; Dunny et al., 2013).

**Diethyl (2-oxo-2-phenylethyl)phosphonate (13)**: viscous oil (82%); ^1^H NMR (400 MHz, CDCl_3_): *δ* = 8.04 (d, *J* = 8.2 Hz, 2H), 7.58 (t, *J* = 8.2 Hz, 1H), 7.49 (t, *J* = 8.2 Hz, 2H), 4.1–4.15 (m, 4H), 3.67 (d, *J* = 23.1 Hz, 2H), and 1.25 (t, *J* = 7.2 Hz, 6H); ^13^C NMR (75 MHz, CDCl_3_): *δ* = 192.0 (d, *J* = 6.7 Hz), 137.2, 134.4, 130.0, 129.4, 62.6 (d, *J* = 6.6 Hz), 38.5 (d, *J* = 129.1 Hz), and 16.6 (d, *J* = 6.2 Hz); ESI–MS (m/z): 256; found 257 [M + H].^+^

**Diethyl (2-(4-methoxyphenyl)-2-oxoethyl)phosphonate (14)**: viscous oil (84%); ^1^H NMR (300 MHz, CDCl_3_): *δ* = 8.01 (d, *J* = 9.0 Hz, 2H), 7.54 (t, *J* = 9. 0 Hz, 1H), 7.46 (t, *J* = 8.2 Hz, 2H), 4.02–4.08 (m, 4H), 3.62 (d, *J* = 22.8 Hz, 2H), and 1.24 (t, *J* = 6.8 Hz, 6H); ESI–MS (m/z): 286; found 287 [M + H].^+^

**Diethyl (2-(3,5-dimethoxyphenyl)-2-oxoethyl)phosphonate (15)**: viscous oil (85%); ^1^H NMR (400 MHz; CDCl_3_), 7.62 (d, *J* = 2.7 Hz, 2H), 6.78 (d, *J* = 2.6 Hz, 1H), 4.2–4.10 (m, 4H), 3.89 (s, 6H), 3.64 (d, *J* = 23.1 Hz, 2H), and 1.29 (t, *J* = 7.2 Hz, 6H); ESI–MS (m/z): 316; found 17 [M + H].^+^

**Diethyl (2-oxo-2-(3,4,5-trimethoxyphenyl)ethyl)phosphonate (16)**: viscous oil (80%) ^1^H NMR (400 MHz; CDCl_3_), 7.58 (s, 2H), 4.2–4.12 (m, 4H), 3.89 (s, 6H), 3.87 (s, 3H), 3.62 (d, *J* = 23.4 Hz, 2H), and 1.25 (t, *J* = 7.1 Hz, 6H); ESI–MS (m/z): 346; found 347 [M + H].^+^

#### 9.1.3 General procedure for the synthesis of benzocoumarin–chalcone hybrids (26–37)

A volume of 6 mmol of NaOMe was added dropwise to a solution of the respective phosphonates (**13–18**, 2 mmol**)** in dry DMF (5 mL) at 0°C. The resulting mixture was stirred at the same temperature for 1 h, followed by the dropwise addition of benzocoumarin-4-carbaldehyde (2**4–25**, 2.0 mmol) in DMF (5 mL), and the reaction mixture was allowed to stir at room temperature for an additional 4 h. On the completion of the reaction, the reaction mixture was quenched with ice water and allowed to stand for 2 h. The solid that precipitated out was filtered, washed with Et_2_O, and crystallized from hot EtOAc to provide the desired benzocoumarin–chalcone hybrid compounds (**26–37**) as pure compounds.

**(E)-4-(3-(3,5-Dimethoxyphenyl)-3-oxoprop-1-en-1-yl)-2H-benzo[h]chromen-2-one (26)**: light yellow solid (95%); mp272–273°C; Anal. (%) for C_24_H_18_O_5_: Calcd., C, 74.60; H, 4.70; found, C, 74.78; H, 4.69; IR (KBr) ν/cm^-1^; ^1^H NMR (300 MHz, CDCl_3_+ DMSO-*d*_6_) *δ* 8.54 (brs, 1H), 8.12 (d, *J* = 15.2 Hz, 1H), 7.92 (brs, 1H), 7.81–7.76 (m, 3H), 7.69–7.67 (m, 2H), 7.22 (s, 2H), 6.89 (s, 1H), 6.73 (s, 1H), and 3.88 (s, 6H); ^13^C NMR (CDCl_3,_ 75 MHz) *δ* 188.4, 161.1, 160.6, 151.2, 149.8, 138.8, 136.8, 135.0, 130.0, 129.1, 127.7, 124.6, 123.2, 122.7, 120.1, 112.4, 106.5, 106.1, and 55.7; ESI–MS: (m/z); 386, found [M + H ]^+^ 387; HRMS-ESI: C_24_H_19_O_5_ [M + H]^+^calcd 387.1232, found 387.1343.

**(E)-4-(3-(4-Methoxyphenyl)-3-oxoprop-1-enyl)-2*H*-benzo[*h*]chromen-2-one (27)**: light yellow solid (93%); mp 255–252°C; IR (KBr) 3040, 1704, 1574, 1465, 1235, 1120, and 812 ν/cm^-1^, ^1^H NMR (300 MHz, CDCl_3_) *δ* 8.64–8.61 (m, 1H), 8.19–8.08 (m, 3H), 7.92–7.91 (m, 1H), 7.77–7.68 (m, 5H), 7.05 (d, *J* = 8.7 Hz, 2H), 6.78 (s, 1H), and 3.94 (s, 3H); ^13^C NMR (CDCl_3,_ 75 MHz) *δ* 186.9, 164.6, 160.7, 151.1, 150.0, 135.9, 135.0, 131.2, 129.9, 129.0, 127.7,127.4, 124.6, 123.2, 122.7, 120.2, 114.2, 113.5,112.2, and 55.6; ESI–MS: (m/z); 356, found [M + H ]^+^ 357; HRMS-ESI: C_23_H_17_O_4_ [M + H]^+^calcd 357.1127, found 357.1132.

**(E)-7-Methoxy-4-(3-(4-methoxyphenyl)-3-oxoprop-1-enyl)-2*H*-benzo[*h*]chromen-2-one (28)**: light yellow solid (92%); mp 236–237°C; Anal. (%) for C_24_H_18_O_5_: Calcd., C, 74.60; H, 4.70; found, C, 74.44; H, 4.75; IR (KBr) 1730, 1604, 1386, 1262, 1177, and 1052 ν/cm^-1^, ^1^H NMR (300 MHz, TFA-*d*_1_) *δ* 8.27–8.16 (m, 5H), 7.92 (d, *J* = 15.5 Hz, 1H), 7.73 (d, *J* = 8.9 Hz, 1H), 7.6 (s, 1H), 7.15–7.13 (m, 4H), 4.06 (s, 3H), and 4.01 (s, 3H); ^13^C NMR (TFA-*d*_1,_ 75 MHz) *δ* 165.2, 154.9, 153.3, 136.5, 132.2, 130.9, 128.6, 128.2, 127.4, 123.4, 120.4, 117.8, 116.5, 115.2, 114.3, 114.1, 112.8, 111.5, 109.6, 108.7, 55.0, and 54.6; ESI–MS: (m/z); 386, found [M + H ]^+^ 387; HRMS-ESI: C_24_H_19_O_5_ [M + H]^+^calcd 387.1232, found 387.1338.

**(E)-7-Methoxy-4-(3-oxo-3-phenylprop-1-en-1-yl)-2H-benzo[h]chromen-2-one (29)**: light yellow solid; yield: 92%, mp 180–182°C; ^1^H NMR (300 MHz, CDCl_3_) *δ* 8.28–8.24 (m, 2H), 7.89 (d, *J* = 8.8 Hz, 1H), 7.77–7.66 (m, 3H), 7.58–7.49 (m, 4H), 7.41 (d, *J* = 15.9 Hz, 1H),7.12 (d, *J* = 7.7 Hz, 1H), 6.81 (s, 1H), and 4.04 (s, 3H); ^13^C NMR (CDCl_3,_ 75 MHz) *δ* 188.6, 162.1, 156.21, 152.2, 151.4,138.4, 136.2, 128.4, 129.1, 128.6, 127.8, 126.9, 124.8, 121.2, 119.6, 118.8, 114.6, 114.6, 110.2, 106.4, and 55.8; ESI–MS: (m/z); 328, found [M + H ]^+^ 329; HRMS-ESI: [M + H]^+^ for C_23_H_17_O_4_ calcd 357.1127, found 357.1119.

**(E)-4-(3-(3,5-Dimethoxyphenyl)-3-oxoprop-1-en-1-yl)-7-methoxy-2H-benzo[h]chromen-2-one (30)**: light yellow solid (97%); mp 258–259°C; Anal. (%) for C_25_H_20_O_6_: Calcd., C, 72.11; H, 4.84; found, C, 72.24; H, 4.82; IR (KBr), 2952, 1728, 1668,1578, 1458,1352, and 1245 ν/cm^-1^, ^1^H NMR (300 MHz, TFA-*d*_1_) *δ* 8.14–8.09 (m, 3H), 7.88 (d, *J* = 7.8 Hz, 1H), 7.70–7.50 (m, 3H), 7.44 (s, 1H), 6.98 (d, *J* = 8.5 Hz, 2H), 6.81(s 1H), 3.88 (s, 3H), and 3.80 (s, 6H); ^13^C NMR (TFA-*d*_1,_ 75 MHz) *δ* 193.0, 160.1,154.8, 153.0, 150.2, 137.8, 137.5, 130.6, 128.1, 123.4, 120.4, 117.8, 116.6, 115.2, 109.9, and 55.1, ESI–MS: (m/z); 416, found [M + H ]^+^ 417; HRMS-ESI: C_25_H_21_O_6_ [M + H]^+^calcd 417.1338, found 417.1349.

**(E)-4-(3-Oxo-3-(3,4,5-trimethoxyphenyl)prop-1-enyl)-2*H*-benzo[*h*]chromen-2-one (31)**: light yellow solid (97%); mp 266–267°C; Anal. (%) for C_25_H_20_O_6_: Calcd., C, 72.11; H, 4.84; found, C, 72.29; H, 4.80; IR (KBr) 3064, 1709,1613, 1468, 1377, 1226, and 1078. 967 ν/cm^-1^, ^1^H NMR (300 MHz, CDCl_3_) *δ* 8.58–8.55 (m, 1H), 8.16 (d, *J* = 15.3 Hz, 1H), 7.90–7.88 (m, 1H), 7.73–7.66 (m, 5H), 7.35 (s, 2H), 6.79 (s, 1H), and 3.99 (s, 9H); ^13^C NMR (CDCl_3,_ 75 MHz) *δ* 187.3, 160.7, 153.3, 151.1, 149.8, 143.3, 136.5, 135.0, 132.1, 129.6, 129.1, 127.7,127.4, 124.6, 123.2, 122.6, 120.0, 113.4, 112.3, 106.2, 61.0, and 56.4; ESI–MS: (m/z); 416, found [M + H ]^+^ 417; HRMS-ESI: C_25_H_21_O_6_ [M + H]^+^calcd 417.1338, found 417.1344.

**(E)-4-(3-Oxo-3-phenylprop-1-enyl)-2*H*-benzo[*h*]chromen-2-one (32)**: light yellow (95%) solid; mp 245–246°C; Anal. (%) for C_22_H_14_O_3_: Calcd., C, 80.97; H, 4.32; found, C, 80.78; H, 4.36; IR (KBr) 3434, 2940, 1709, 1584, 1460, 1237, 1126, and 814 ν/cm^-1^, ^1^H NMR (300 MHz, CDCl_3_) *δ* 8.61–8.59 (m, 1H), 8.20–8.08 (m, 3H), 7.91–7.90 (m, 1H), 7.75–7.68 (m, 6H), 7.61–7.56 (m, 2H), and 6.77 (s, 1H); ^13^C NMR (CDCl_3,_ 75 MHz) *δ* 188.7, 160.6, 151.1, 149.8, 136.9, 136.7, 135.0, 133.8, 129.9, 129.1, 128.7, 127.7, 127.4, 124.6, 123.2, 122.7, 120.1, 113.3, and 112.4; ESI–MS: (m/z); 326, found [M + H ]^+^ 327; HRMS-ESI: C_22_H_15_O_3_ [M + H]^+^calcd 327.1021, found 327.1032.

**(E)-7-Methoxy-4-(3-oxo-3-(3,4,5-trimethoxyphenyl)prop-1-enyl)-2*H*-benzo[*h*] chromen-2-one (33)**: light yellow solid (94%); mp > 260°C; Anal. (%) for C_26_H_22_O_7_: Calcd., C, 69.95; H, 4.97; found, C, 69.78; H, 4.96; IR (KBr) 3434, 2941, 1733, 1663,1588, 1461,1344, and 1241 ν/cm^-1^, ^1^H NMR (300 MHz, CDCl_3_) *δ* 8.18–8.10 (m, 3H), 7.74–7.67 (m, 2H), 7.60–7.53 (m, 1H), 7.35 (s, 2H), 7.02 (d, *J* = 7.7 Hz, 1H), 6.82 (s, 1H), 4.04 (s, 3H), and 3.99 (s, 9H); ^13^C NMR (CDCl_3_, 75 MHz) *δ* 187.4, 161.2, 155.1, 153.3, 150.7, 150.0, 143.3, 136.5, 132.1, 129.6, 127.8, 127.0, 124.2, 119.1, 119.0, 114.4, 113.8, 112.3, 107.1, 106.2, 61.40, 56.4, and 55.7; ESI–MS: (m/z); 446, found [M + H ]^+^ 447; HRMS-ESI: [M + H]^+^ C_26_H_23_O_7_ [M + H]^+^calcd 447.1444, found 447.1448.

### 9.2 Biology

#### 9.2.1 Cell culture

MCF-7, MDA-MB231, and HEK293 cell lines were purchased from ATCC (Manassas, VA, United States). Ishikawa cell lines were obtained from the European Collection of Cell Cultures. They were maintained in Dulbecco’s modified Eagle medium (DMEM) supplemented with 10% fetal bovine serum (FBS). Cells were cultured at 37°C and 5% CO_2_. Before the experiments, cells were cultured in phenol red-free DMEM supplemented with 10% charcoal stripped fetal bovine serum.

#### 9.2.2 Cell proliferation assay (MTT assay) in cancer cell lines and normal cell lines

The synthesized hybrid compounds were tested for their anti-cancer activity using the MTT reduction assay on 2.5 × 10^3^ cells per well that were seeded in 100 µL DMEM supplemented with 10% FBS in each well of 96-well microculture plates. After seeding, the cells were incubated for 24 h at 37°C in a CO_2_ incubator. The required concentrations of the compounds were achieved by diluting the solutions in the culture medium. The media was removed from the wells after 48 h, and 100 µL of MTT (0.5 mg/mL) was added to each well. The plates were then incubated for an additional 3 h. After carefully removing the supernatant from each well, the formazan crystals were dissolved in 100 µL of DMSO. The absorbance was then recorded at a wavelength of 540 nm (Saquib et al., 2021; Gupta et al., 2022; Iqbal et al., 2023)

### 9.3 Molecular docking studies

Molecular docking is used to predict the possible binding abilities of ligands within the pockets of amino acid residues of target protein receptors. To find out the possible binding modes, the calculation of docking scores was carried out for all synthesized compounds, **26–37**, against four cell proliferating proteins, namely, NF-kβ, TNF-α, MAPK, and JNK, using the Glide module of Maestro version 12.6.144 (Schrödinger 2020–4 LCC, New York, United States) software. The chemical structures of compounds, **26**–**37**, were drawn using ChemDraw Professional 15.0 software, and the 2D structures were saved in the mol format (Table 1). Ligands were prepared using the LigPrep module of Maestro version 12.6.144 (Schrödinger 2020–4 LCC, New York, United States) software. The minimization of all ligands corrected the bond length and angle, and generated the possible conformers by using the OPLS force field. All the feasible protonation and ionization states were itemized for each compound using Epik pH 7.0±2.0 and then saved in the Maestro format (Fadaka et al., 2022). The crystal structure of protein receptors, namely, TNF-α, (PDB ID 6X81), JNK (1JNK), MAPK (1A9U), and NF-kB (1SVC), having the resolution of 2.81 Å, were obtained from the RCSB PDB database (https://www.rcsb.org/) and then prepared by using the protein preparation wizard module of Maestro version 12.6.144 (Schrödinger 2020–4 LCC, New York, United States) software. The refinement of the protein structure was carried out by the removal of the heteroatom and water molecules, followed by the assignment of the bond order with added hydrogen atoms using the OPLS force field and minimized the receptor (Pattar et al., 2020). In order to identify the top-ranked potential receptor-binding sites in protein, the active sites were predicted for ligand binding to the receptor using the sitemap module of Maestro version 12.6.144 (Schrödinger 2020–4 LCC, New York, United States) software (Halgren, 2009). The grid box was centroid over the active site of the protein, which was predicted by the sitemap with the van der Waals scaling factor of 1.0 Å, and the partial charge cut-off was 0.25 Å (Sharma et al., 2016). For molecular docking, the softened potential was considered by the van der Waal radii scaling factor of 0.80 Å with a 0.15-Å partial charge cut-off for non-polar parts of ligands. After docking, we saved the best pose of the ligand–protein complex based on the docking scores (Ansari et al., 2022).

### 9.4 Molecular dynamic simulation

The molecular dynamic simulation study was conducted to check the stability of the ligand–protein complex. The MD simulation was implemented using the Desmond module of Maestro version 12.6.144 (Schrödinger 2020–4 LCC, New York, United States) software. The best pose of the docked ligand–protein complex was immersed into the TIP3P water solvent model and generated the orthorhombic boundary with the shape size of 10 Å × 10 Å X10 Å. The system was neutralized by Na^+^/Cl^−^ ions at 0.15 M salt concentration, and the whole system was minimized by applying the OPLS3 force field. The MD simulation was performed in an NPT ensemble at 300 K and 1.013 bar pressure over the 100-ns simulation. To maintain the temperature and pressure, we used the Nose–Hoover chain thermostat and Martyna–Tobias–Klein barostat method, respectively. The RESPA (reversible reference system propagator algorithm) integrator was used with time to accelerate the simulation of each 2.0 fs step with the PME (particle–mesh Ewald) method for the calculation of the long-range electrostatic interaction. Finally, the energy was evaluated at every 100.0 ps interval after analyzing the simulation trajectory (Ansari et al., 2022).

### 9.5 Pharmacological properties

The pharmacological properties of **26**–**37** compounds were assessed using the QikProp v6.8 tool of Maestro 12.6.144 (Schrödinger 2020–4 LCC, New York, United States) software. The QikProp v6.8 tool analyzes a number of significant factors, such as molecular weight (MW), hydrogen bond donor (HBD), hydrogen bond acceptor (HBA), polar surface area (PSA), lipophilicity (LogP), aqueous solubility (LogS), Caco cell permeability, MSCK permeability, human serum albumin (HSA), and human oral absorption (HOA), that are useful in predicting drug-like candidate properties (Mohan et al., 2022)

## Data Availability

The original contributions presented in the study are included in the article/Supplementary material; further inquiries can be directed to the corresponding authors.
